# SARS-CoV-2 screening in cancer outpatients during the second wave of the COVID-19 pandemic: Conclusions for crisis response at a high-volume oncology center

**DOI:** 10.1007/s00508-021-01927-7

**Published:** 2021-08-19

**Authors:** Julia M. Berger, Margaretha Gansterer, Wolfgang Trutschnig, Arne C. Bathke, Robert Strassl, Wolfgang Lamm, Markus Raderer, Matthias Preusser, Anna S. Berghoff

**Affiliations:** 1 Division of Oncology, Department of Medicine I, Medical University of Vienna, Waehringer Guertel 18–20, 1090 Vienna, Austria; 2 Faculty of Management and Economics, University of Klagenfurt, Klagenfurt, Austria; 3 Faculty of Natural Sciences and Intelligent Data Analytics Lab Salzburg, University of Salzburg, Salzburg, Austria; 4 Department of Laboratory Medicine, Division of Clinical Virology, Medical University of Vienna, Vienna, Austria

**Keywords:** Polymerase chain reaction (PCR), Asymptomatic infection, Safety measure, Cancer patient care, Testing strategy

## Abstract

**Background:**

During the second wave of the coronavirus disease 2019 (COVID-19) pandemic Austria suffered one of the highest severe acute respiratory syndrome coronavirus 2 (SARS-CoV-2) rates worldwide. We report performance parameters of a SARS-CoV‑2 screening program established for cancer outpatients at our center.

**Methods:**

Institutional policy recommended routine biweekly SARS-CoV‑2 testing. Adherence to the testing recommendation during the second wave of the COVID-19 pandemic between 1 October and 30 November 2020 was analyzed. The SARS-CoV‑2 infection rate during first wave period (21 March to 4 May 2020) was compared to the one during second wave.

**Results:**

A total of 1577 cancer patients were seen at our outpatient clinic during the second wave. In 1079/1577 (68.4%) patients, at least 1 SARS-CoV2 test was performed. Overall 2833 tests were performed, 23/1577 (1.5%, 95% confidence interval, CI 1.0–2.2%) patients were tested positive for SARS-CoV‑2, which indicates a significant increase compared to the first wave (4/1016; 0.4%, 95% CI 0.1–1.0%) with an odds ratio of 3.9 (95% CI 1.5–10.1; *p* < 0.005). Patients undergoing active anticancer treatment (172/960; 17.9% not tested) were more likely to have undergone a SARS-CoV‑2 test than patients in follow-up or best supportive care (326/617; 52.8% not tested *p* < 0.001). Furthermore, patients with only 1 visit within 4 weeks were more likely to not have undergone a SARS-CoV‑2 test (386/598; 64.5%) compared to patients with 2 or more visits (112/979; 11.4%; *p* < 0.001). The projected number of patients with undetected SARS-CoV‑2 infection during the study period was 5.

**Conclusion:**

We identified clinical patient parameters influencing SARS-CoV‑2 testing coverage in cancer outpatients. Our data can provide information on generation of standard operating procedures and resource allocation during subsequent infection waves.

## Introduction

The coronavirus disease 2019 (COVID-19) pandemic has caused a global health crisis and caused over 1,532,418 deaths worldwide until 7 December 2020 [1]. Oncology patients were repetitively reported to be at a particular risk with fatality rates of up to 11.4% [2–4]. Potential factors contributing to the higher risk for adverse COVID-19 courses among cancer patients include the high average age, high rate of comorbidities, disease-associated and therapy-induced immunosuppression and unavoidable social contacts during regular therapy and follow-up visits at the hospital [5]; however, besides direct endangerment of cancer patients by severe acute respiratory syndrome coronavirus 2 (SARS-CoV-2) infections, concerns about adverse outcomes associated with disruptions in oncological care due to COVID-19 have emerged [6, 7]. Therefore, continued administration of anti-cancer therapies has been defined as a priority by oncological societies and cancer centers around the world [8, 9].

At our large tertiary care center of medical oncology with approximately 40,000 patient contacts per year, we have rapidly implemented strict safety measures during the first wave of the COVID-19 pandemic in Spring 2020. We could show that these safety precautions resulted in low rates of detectable SARS-CoV‑2 infections among our patients and anti-SARS-CoV‑2 antibodies among our patients and staff that allowed continued patient care and therapy at our center [10, 11].

The second wave of the COVID-19 pandemic in the fall of 2020 hit Austria particularly hard as Austria was among the countries with the highest 7‑day incidence rates worldwide with 565 new infections per 100,000 on 12 November 2020 [12]. In order to protect the well-being of our patients and staff as well as the functionality of our clinical service, we implemented strict safety measures during the second wave based on our experience during the first wave. Although many institutions refrained from regular SARS-CoV‑2 testing of patients owing to logistic restraints, the safety measures implemented at our institution included a recommendation for biweekly testing of all patients for SARS-CoV‑2 RNA using nasopharyngeal swabs and polymerase chain reaction (PCR).

Here, we analyzed performance parameters of this SARS-CoV‑2 screening program at our center during the second wave. We aim to provide an information basis for optimization of standard operating procedures and resource allocation for crisis response during potential subsequent infection waves of the COVID-19 pandemic.

## Methods

This study was approved by the ethics committee of the Medical University of Vienna (vote number 2485 of 2020).

### Patient cohort

All patients treated at the outpatient department of the Division of Oncology, Department of Medicine 1, Medical University of Vienna between 1 October and 30 November 2020 were included in this retrospective analysis. During this period, only outpatients without COVID-19 symptoms were permitted access to our department and our institutional policy recommended routine biweekly real-time polymerase chain reaction-based SARS-CoV‑2 testing. Testing was performed by PCR test only. Further safety measurements and the date of implementation are listed in Table 1. A patient cohort treated at our department and tested for SARS-CoV‑2 during the first wave of the COVID-19 pandemic between 21 March and 4 May 2020 was available for comparison [10].

**Table 1:** Safety measures implemented by the General Hospital of Vienna and the Division of Oncology

Measure	Date of implementation/time period
Formation of hospital staff cohorts – 1st wave	15 March–31 May 2020
– 2nd wave	02 November–07 December 2020
Separate access for patients with structured triage by healthcare professionals	16 March 2020—ongoing
Provision of masks and protective gear to hospital staff and patients	16 March 2020—ongoing
Implementation of hygiene recommendation to test all patients for SARS-CoV‑2	23 March 2020
Implementation of a 2-week basis for SARS-CoV‑2 retesting – Testing at the visiting unit	21 March 2020
– Testing at central testing unit	05 August 2020
Implementation of the hygiene recommendation to test all patients the day before a visit	23 March 2020

### SARS-CoV-2 testing

Testing for SARS-CoV‑2 viral RNA was exclusively performed by nasal or pharyngeal swabs and real-time polymerase chain reaction (RT-PCR). RT-PCR analysis was either performed using an european conformity in-vitro diagnostics (CE/IVD) validated workflow (Cobas SARS-CoV‑2 assay on the Roche Cobas 6800 platform [Roche, Basel, Switzerland]; Abbott RealTime SARS-CoV‑2 assay on the Abbott m2000 platform [Abbott, Chicago, IL, USA]) or using a validated RT-PCR workflow according to Corman et al. [13]. The rate of false positive results is estimated at 0.04% whereas the rate of false positive tests is estimated at 0.0–1.0% [14–16]. All analyses were carried out at the Department of Laboratory Medicine, Division of Clinical Virology, Medical University of Vienna, Vienna, Austria. Comparability of the results of all test methods was demonstrated by participating in the international quality control ring trials [17].

### Statistical analysis

The first wave was defined from 21 March to 10 May 2020. The second wave was defined from 1 October to 30 November 2020. Data from the general Austrian population was available from the Federal Ministry of Social Affairs, Health, Care and Consumer Protection and the AGES (Austrian Agency for Health and Food Safety) [12, 18]. For comparison of the increase in SARS-CoV‑2 positivity, tests of each individual within the testing period (first or second wave) were used. For individuals with more than 5 tests during 1 of the 2 time periods, 5 tests were randomly selected. Statistical analysis was performed using the SPSS V.27 software package (SPSS, Chicago, IL, USA). A two-sided *P* value of 0.05 was defined as significance threshold. Studentʼs t‑test, and χ^2^-test were applied as indicated. Shapiro-Wilk test was used to test for normal distribution. We compared prevalence between cohorts using the estimated odds ratio and Fisher’s exact test. For estimating the number of undetected SARS-CoV‑2 infections we used the observed prevalence in patients during the second wave.

## Results

### Patient characteristics

A total of 1577 individual patients were included in the present analysis (median age 63 years; 57.9% female; 42.1% male). The most common cancer diagnoses were breast cancer (377/1577; 23.9%), lung cancer (238/1577; 15.1%), colorectal cancer (132/1577; 8.4%), sarcoma (123/1577; 7.8%), glioma (112/1577; 7.3%), head and neck cancer (101/1577; 6.4%) and lymphoma (94/1577; 6%). A total of 617/1577 (39.1%) patients were not undergoing active anticancer treatment but either in follow-up, best supportive care or wait and see strategy. The most common forms of treatment were chemotherapy (380/1577; 24.1%), followed by targeted therapy (345/1577; 21.9%), immunotherapy (111/1577; 7%) or a combination of the above. Of the patients 725/1577 (46%) were treated with palliative intent and 209/1577 (13.3%) patients in an adjuvant setting. Patient characteristics are given in Table 2.

**Table 2:** Patient characteristics

Characteristic	Cancer cohort (*n* = 1577)	
	*n*	%
*Gender*		
Male	664	42.1%
Female	913	57.9%
*Age at SARS-CoV‑2 testing*		
Median, years (range)	63 (18–93)	
*Anti-neoplastic treatment*		
None	617	39.1%
Chemotherapy	380	24.1%
Targeted therapy	345	21.9%
Immunotherapy	111	7.0%
Chemotherapy and targeted therapy	84	5.3%
Chemotherapy and immunotherapy	32	2.0
Targeted therapy and immunotherapy	8	0.5
*Metastases*		
Present	802	50.9
Absent	775	49.1
*Number of hospital visits per 4 weeks*		
Median (range)	2 (1–12)	
*Number of SARS-CoV‑2 test per 4 weeks*		
Median (range)	1 (0–11)	
*SARS-CoV‑2 test performed*		
Yes	1.079	68.4
No	498	31.6
*SARS-CoV‑2 infection detected*		
Yes	23	1.5
No	1.554	98.5

### SARS-CoV-2 testing rates

**Fig. 1:**
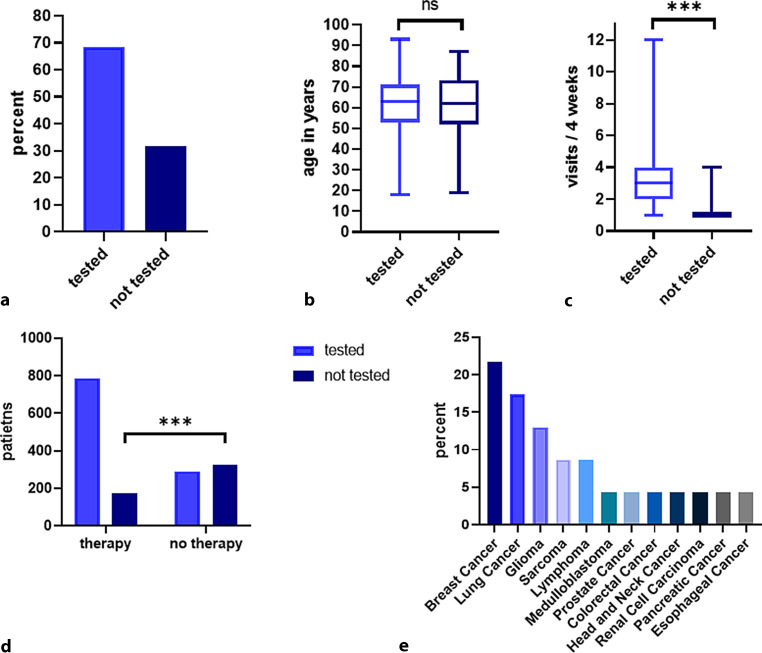
**a** Patients according to performed test; **b** median age in tested and not tested patients; **c** median number of visits of tested and not tested patients; **d** therapy status in tested and not tested patients; **e** distribution of patients with SARS CoV‑2 infection according to primary tumor. *ns* not significant, *** p < 0.001

During the second wave period, a total of 2833 SRAS-CoV‑2 tests were performed in 1577 patients and 1079/1577 (68.4%) patients were tested at least once during the observation period (Fig. 1a). Overall, the range of tests per patient was 0–11 with a median of 1 test per patient. Age between patients tested versus the ones without test was not different (median 63 years versus 62 years; *p* > 0.05; Fig. 1b). Patients with only one visit within a time period of 4 weeks were more likely to not have undergone a SARS-CoV‑2 test (386/598; 64.5%) compared to patients with two or more visits (112/979; 11.4%; *p* < 0.001; Fig. 1c). A significantly higher number of patients undergoing active anti-cancer therapy (326/617; 52.8%) were tested for SARS-CoV‑2 as compared to patients not undergoing active anti-cancer therapy (172/960; 17.9%) at the time of presentation to our department (*p* < 0.001; Fig. 1d).

### SARS-CoV-2 detection rates

During the second wave period, SARS-CoV‑2 was detected in 23/1577 (1.5%, 95%-CI: 1.0–2.2%) patients (male 47.8%; female 52.2%) at our department. Oncological diagnoses in these patients included breast cancer (5/23; 21.7%), lung cancer (4/23; 17.4%), glioma (3/23; 13%), sarcoma and lymphoma (2/23; 8.7% each), medulloblastoma, prostate cancer, colorectal cancer, head and neck cancer, renal cell carcinoma, pancreatic cancer and esophageal cancer (1/23; 4.3% each) (Fig. 1e). Of the patients 21/23 (91.9%) developed symptoms associated with COVID-19 during the infection, 1/23 (4.3%) fatal and 2/23 (8.7%) severe disease courses were observed. All patients had at least one PCR test result with a cycle threshold (Ct) below 30.

**Fig. 2:**
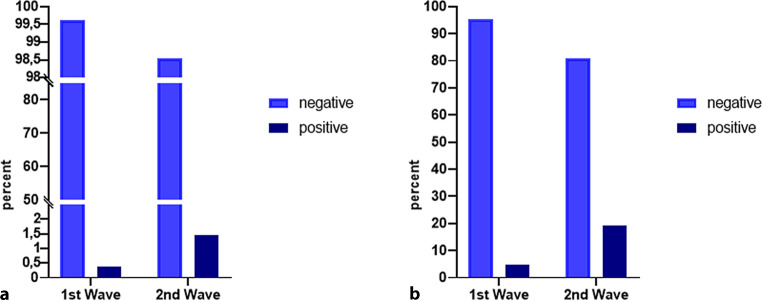
Rate of individuals tested positive on SARS-CoV‑2 in percent **a** cancer cohort; **b** Austrian population

The detection rate of 23/1577 (1.5%, 95% confidence interval, CI 1.0–2.2%) indicates a significant increase of infections compared to the first wave in spring 2020 (4/1016; 0.4%, 95% CI 0.1–1.0%). Among patients at our institution, the odds ratio for comparison of positive tests in the second versus the first wave was 3.9 (95% CI 1.5–10.1; *p* < 0.005). (Fig. 2a).

The projected number of patients with undetected SARS-CoV‑2 infection during the second wave at our department was five.

In the Austrian population 12,717/266,354 (4.8%) individuals were tested positive for SARS-CoV‑2 in the first wave period compared to 238,628/1,241,703 (19.2%) individuals during the second wave period. In the Austrian population, the odds ratio for comparison of positively tested patients in the second versus the first wave was 4.0 (95% CI 4.0–4.1; *p* < 0.001). (Fig. 2b) Therefore, both in the general Austrian population as well as in the cancer cohort the odds to be infected with SARS-CoV‑2 in the second wave were approximately 4 times higher than in the first wave.

## Discussion

SARS-CoV‑2 testing is essential to prevent viral transmission and curb the on-going pandemic by early identification and isolation of infected individuals. Due to the incubation period of 3–6 days, asymptomatic virus carriers are major contributors to the overall viral spread, particularly in the setting of healthcare facilities [19–22]. Consequently, focused testing in healthcare facilities is recommended to prevent nosocomial COVID-19 infections and maximize safety of patients and staff [19]. In the present data we observed comparable increases in SARS-CoV‑2 infections in the cancer cohort as in the general Austrian population. We achieved a 68.4% testing coverage of cancer outpatients treated at our department during the second wave of the COVID-19 pandemic between October 1st and November 30th using an institutional policy recommending biweekly SARS-CoV‑2 testing. Our strategy is based on our previously reported experience [10] and differs from other centers that perform SARS-CoV‑2 testing only in selected patients [21, 23–25].

Overall, our data indicate that routine PCR-based testing is feasible at a large department of medical oncology with over 3000 patient contacts per month; however, perfect test coverage was not achieved, leading to a projected number of five undetected virus carriers at our department during the observation period. We report that clinical patient parameters apparently influenced SARS-CoV‑2 testing rate in cancer in a high-volume setting. We believe that our data may be useful for adaptation of standard operating procedures and resource allocation in order to optimize protective measures during potential subsequent infection waves of the COVID-19 pandemic.

Patients currently not under active antineoplastic treatment and patients with a lower number of visits were less likely to undergo SARS-CoV‑2 testing. The experience gathered during the observation period indicates that logistical challenges are main contributors to limiting full test coverage of all patients. The infrastructure of our institution did not allow for direct easy access testing during patient presentation at our department but necessitated the referral of patients to a central SARS-CoV‑2 testing unit that services the entire hospital. The implementation of a central testing unit allows efficient resource allocation and standardized testing at a large center; however, it increases the complexity of patient administration for staff members and the number of in-hospital transits and waiting times for individual patients. These factors likely have contributed to our results and need to be taken into account for informed application of safety measures to contain viral spread. As a consequence of our data, we advocate routine extramural testing of patients not undergoing active anticancer therapy just before presentation to a tertiary care oncology service, e.g. at general practitioners or testing facilities for the general public. In-hospital testing should be reserved for patients undergoing active anticancer therapy in order to achieve focused resource allocation. This measure should be suitable to avoid underdetection of SARS-CoV‑2 positive patients in the vulnerable medical oncology center and thus increase patient and staff safety [26].
